# Supplementary material for: Isolation of Thermophilic Bacteria from Extreme Environments in Northern Chile
Source: Microorganisms. 2024 Feb 27;12(3):473. doi: 10.3390/microorganisms12030473 (PMC10972389; doi:10.3390/microorganisms12030473)
Supplement: Supplementary file 1 [file microorganisms-12-00473-s001.zip › microorganisms-2824867-supplementary.pdf]

Supplementary Figures

CJ1

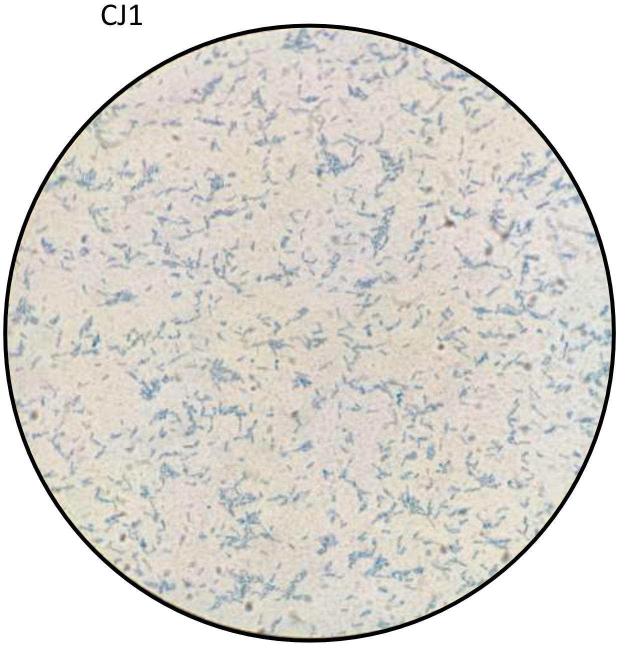

CJ5

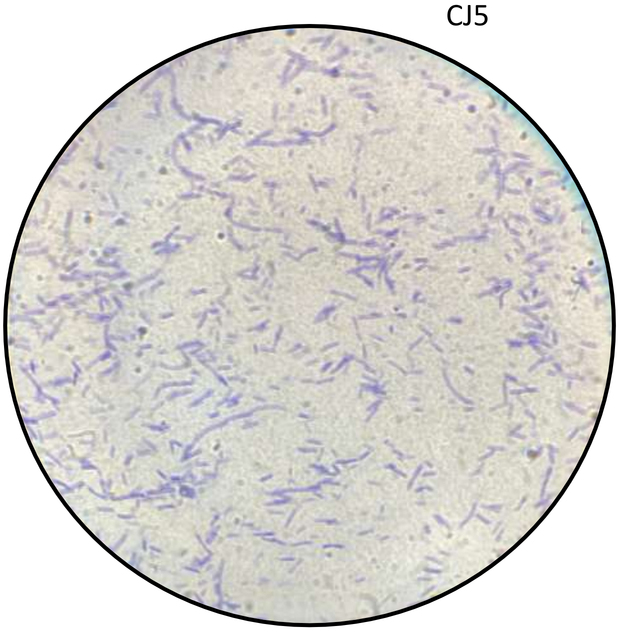

CT1

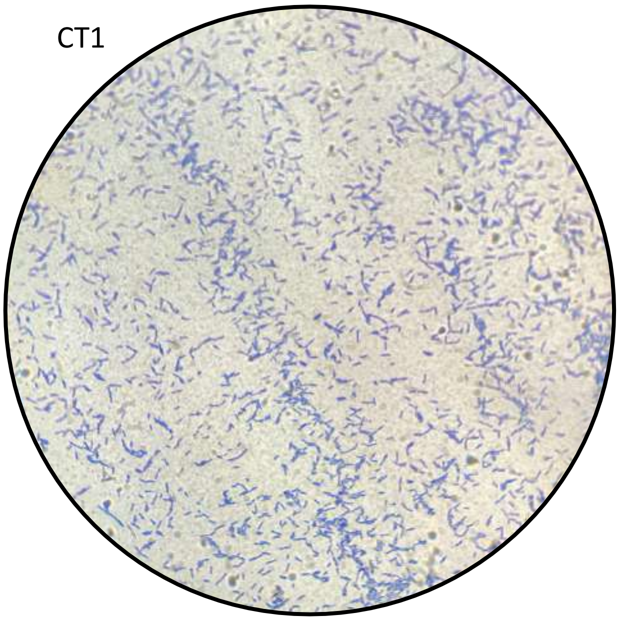

702B

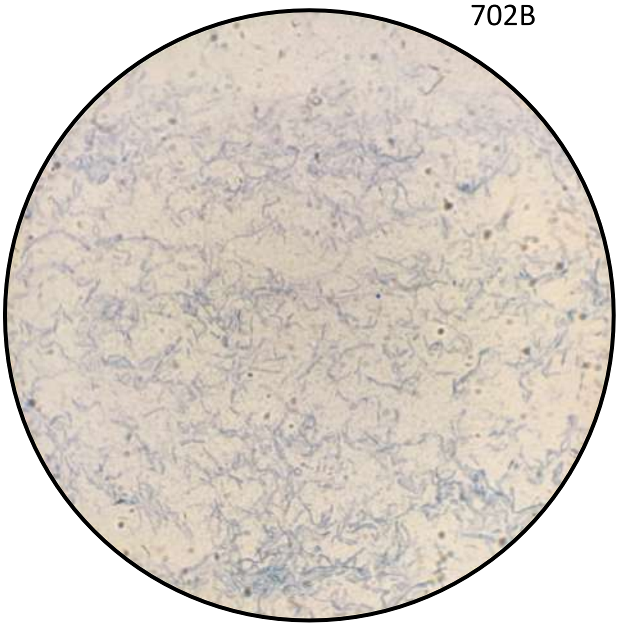

2A55

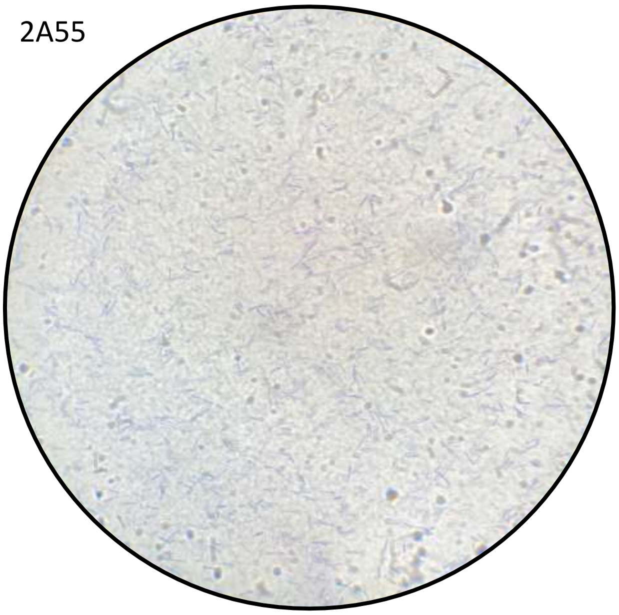

2B55

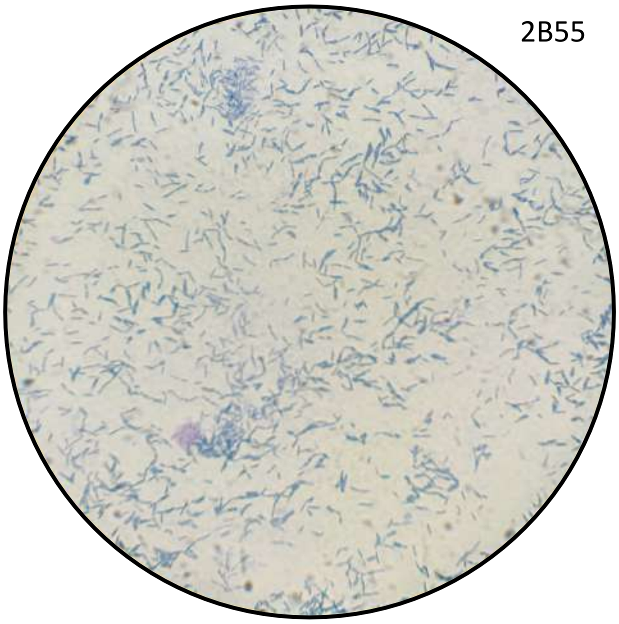

SA

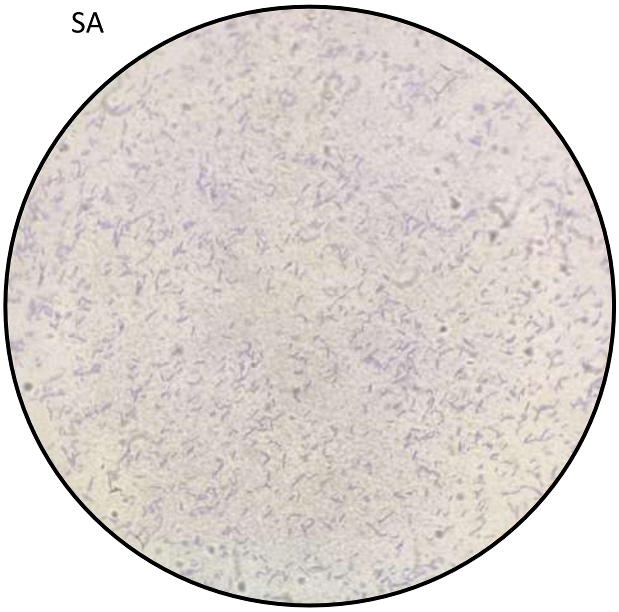

2*55

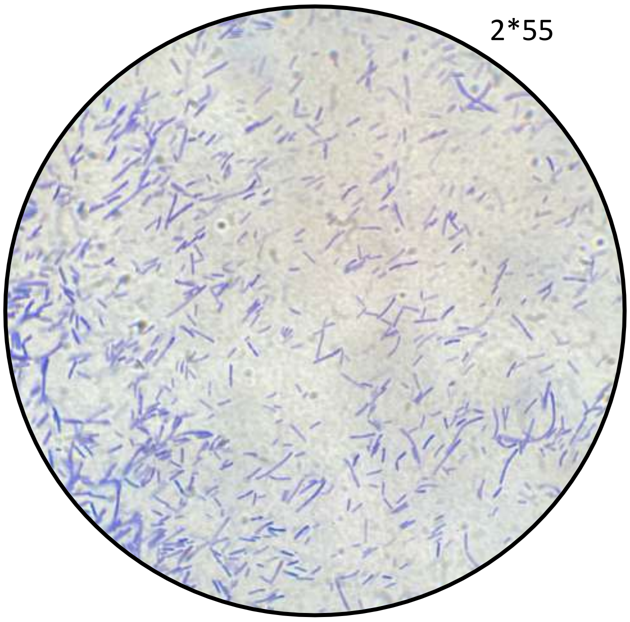

M8

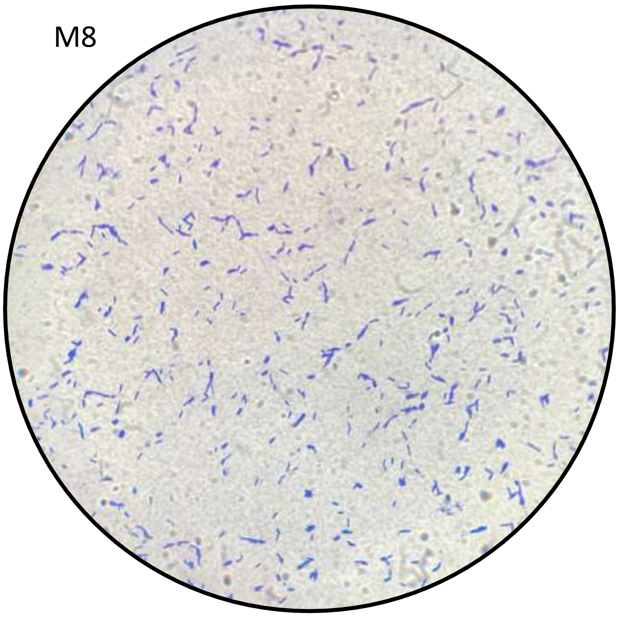

3A55

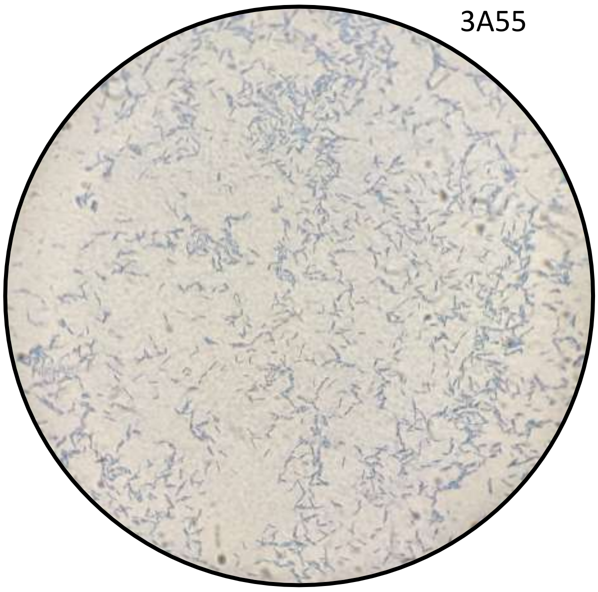

TB4

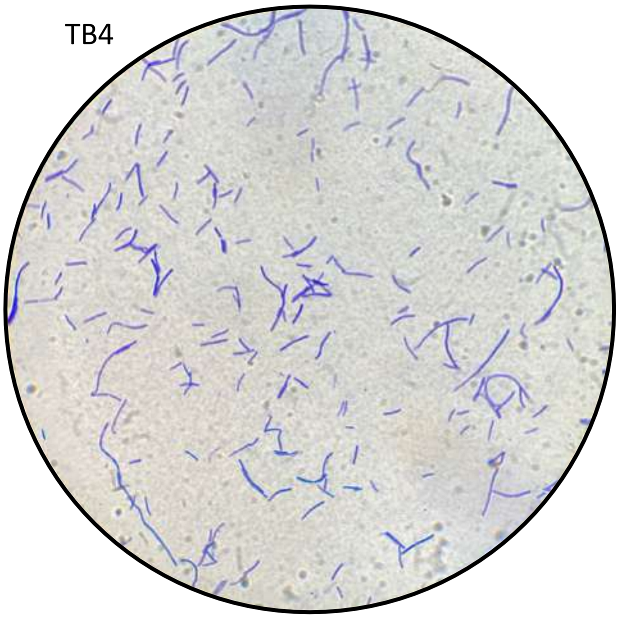

TB5

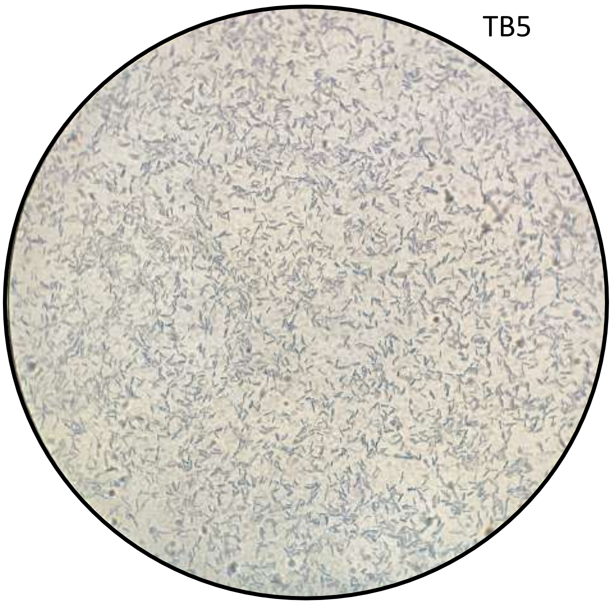

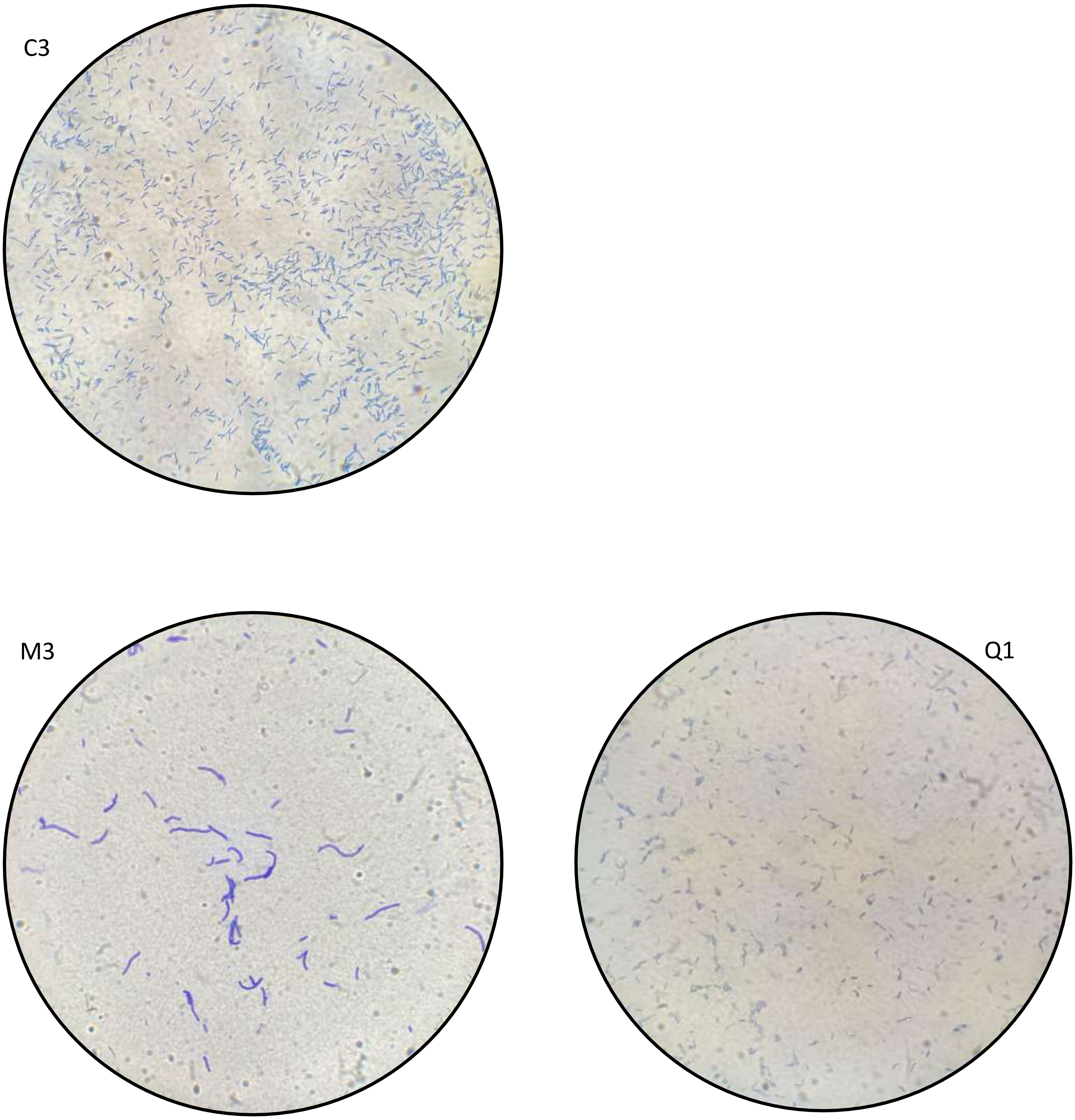

Fig 1S. Gram staining (1,000X) of the isolated strains: CJ1 y CJ5 (*Termas Jurasi*); CT1,702b, 2A55, 2B55, SA, 2*55, M8 and 3A55 (*El Tatio* Geothermal Field); TB4 and TB5 (*Laguna Tebenquiche*); C3 (*Laguna Cejar);* M3 and Q1 (*Quebrada Carrizo*).
